# The burden of disease profile of residents of Nairobi's slums: Results from a Demographic Surveillance System

**DOI:** 10.1186/1478-7954-6-1

**Published:** 2008-03-10

**Authors:** Catherine Kyobutungi, Abdhalah Kasiira Ziraba, Alex Ezeh, Yazoumé Yé

**Affiliations:** 1 African Population & Health Research Center, P.O Box 10787, GPO 00100, Nairobi, Kenya

## Abstract

**Background:**

With increasing urbanization in sub-Saharan Africa and poor economic performance, the growth of slums is unavoidable. About 71% of urban residents in Kenya live in slums. Slums are characteristically unplanned, underserved by social services, and their residents are largely underemployed and poor. Recent research shows that the urban poor fare worse than their rural counterparts on most health indicators, yet much about the health of the urban poor remains unknown. This study aims to quantify the burden of mortality of the residents in two Nairobi slums, using a Burden of Disease approach and data generated from a Demographic Surveillance System.

**Methods:**

Data from the Nairobi Urban Health and Demographic Surveillance System (NUHDSS) collected between January 2003 and December 2005 were analysed. Core demographic events in the NUHDSS including deaths are updated three times a year; cause of death is ascertained by verbal autopsy and cause of death is assigned according to the ICD 10 classification. Years of Life Lost due to premature mortality (YLL) were calculated by multiplying deaths in each subcategory of sex, age group and cause of death, by the Global Burden of Disease standard life expectancy at that age.

**Results:**

The overall mortality burden per capita was 205 YLL/1,000 person years. Children under the age of five years had more than four times the mortality burden of the rest of the population, mostly due to pneumonia and diarrhoeal diseases. Among the population aged five years and above, HIV/AIDS and tuberculosis accounted for about 50% of the mortality burden.

**Conclusion:**

Slum residents in Nairobi have a high mortality burden from preventable and treatable conditions. It is necessary to focus on these vulnerable populations since their health outcomes are comparable to or even worse than the health outcomes of rural dwellers who are often the focus of most interventions.

## Background

Africa is urbanizing at a faster rate than any other region in the world and by 2030, more than half of the population of sub-Saharan Africa will live in urban areas [1]. The rapid growth of urban centres in many sub-Saharan African countries has occurred largely in an environment of poor economic performance and lack of urban planning and regulation. This has resulted in an increase in the number and size of informal settlements or slums in many cities. Current estimates from the UN HABITAT suggest that more than 70% of urban residents in sub-Saharan Africa live in slum or slum-like conditions. In Kenya, this percentage is about 71% [2]. Because of the informal nature of these settlements, they are underserved by health, education, water and sanitation, and garbage collection services.

Apart from lack of social amenities, slums are also characterized by high unemployment, overcrowding, insecurity, greater involvement in risky sexual practices, social fragmentation, and high levels of mobility [3-5]. As a result, slums have become the new hubs of poverty and ill health. Studies from different sub-Saharan African countries have shown that slum residents have worse health indicators than their counterparts in rural areas [5-9]. For example, children under the age of five years living in urban poor areas in Zambia and Malawi had higher mortality than those in rural or peri-urban areas [10,11]. The worse health status of slum children could be explained by the continuous exposure to environmental hazards coupled with lack of social services. Desperate living conditions and lack of livelihood opportunities could predispose residents to risky health-related behaviours such as high alcohol consumption, unsafe sex, smoking and other substance abuse. In the short and medium term, these behaviours may lead to higher mortality from external causes and communicable diseases such as HIV/AIDS, and in the long term – to higher risk of non-communicable diseases.

In order to plan for and address the health needs of a population, information is needed about its health status. However, information for health planning is largely unavailable in most developing countries partly due to lack of vital registration. Other data on health status are often derived from small hospital-based studies; yet most illnesses and deaths occur outside the health care system. Estimating the burden of disease in a population is one way to generate health information for planning. Burden of disease projects have been undertaken and estimates have been made for the whole world [12,13] and world regions including sub-Saharan Africa [12,14]. While these estimates are useful for making national and international policy decisions, they mask inter-country and intra-country differences and may not be relevant for local health planning [15]. The situation is even worse for marginalized populations such as the urban poor, which have hitherto not received much attention in terms of research and service delivery. A Burden of Disease (BoD) approach for district and national planning has been successfully used in Tanzania with a positive impact on childhood mortality [16].

This paper therefore aims to describe the causes and quantify the burden of mortality among residents of two Nairobi slums, using a BoD approach with data generated from a Demographic Surveillance System (DSS).

## Methods

### The Nairobi Urban Health and Demographic Surveillance System

The study was conducted in the demographic surveillance area (DSA) of the Nairobi Urban Health and Demographic Surveillance System (NUHDSS); a member of the INDEPTH Network. The NUHDSS was set up in September 2002 after a pilot phase that covered four slums from August 2000 to April 2002 and a baseline census in August 2002 that covered two slums. The DSA covers large parts of the two slums of Korogocho and Viwandani in Nairobi, Kenya's capital and commercial centre. The two slums are located about 5–10 km from the city centre and occupy an area of 0.45 and 0.52 km^2 ^respectively. The nature of slums underscores their non-permanence, and implicitly justifies the official neglect of these communities in the provision of infrastructure and social services, including water, electricity, health services, and law enforcement. There are very few public health facilities serving the two slum communities, and these are located on the outskirts of the slums and are therefore inaccessible at night due to insecurity. The residents are from over 15 ethnic backgrounds with the majority being Kikuyu (28%), Luhya (24%), Kamba (21%) and Luo (15%). In Viwandani, the population is mainly comprised of labour migrants working in the neighbouring industrial area, while the Korogocho population consists mainly of long-term settlers engaged in the informal sector. Data on core demographic events (birth, death, in-migration and out-migration) are collected and updated every four months during routine DSS rounds.

### Cause of death ascertainment

For all deaths recorded during routine DSS round updates, a detailed verbal autopsy questionnaire is administered to a close relative of the deceased or other credible respondent who has knowledge of the symptoms and any medical assistance received before death [17,18]. Different forms are used for deaths among children under the age of five and for all persons aged five years and above. The former includes a section for deaths among infants <28 days old. Several visits are made to the household in order to ensure that a credible respondent is interviewed. After five such visits or if it is established that the remaining household members are no longer resident in the area, a credible neighbour is interviewed if he/she is willing. In cases where there is no credible household member, close relative or neighbour to be interviewed, the verbal autopsy is coded as missing.

Filled verbal autopsy questionnaires are then reviewed by three independent physicians to derive the most probable cause of death. If two or more physicians agree on a cause of death, it is assigned. If not, the most probable cause of death is assigned after reaching consensus at a meeting of the three physicians. Where disagreements persist, the cause of death is coded as unknown. In cases where the cause cannot be ascertained it is coded as undetermined. Causes of death are classified according to the International Classification of Diseases, 10^th ^Revision (ICD-10) using a modified and shortened code list which can be mapped on the global burden of disease cause list. The list was modified in such a way that uncommon causes of death in the study area are collapsed in broader categories. For example, rare communicable diseases such as Trypanosomiasis are coded as "other specified communicable diseases," since it is not expected that enough cases will appear in the specific disease category for any meaningful analysis.

### Estimation of Years of Life Lost (YLL) due to premature death

All deaths were grouped by sex, age groups (<1, 1–4, five-year age groups thereafter) and cause of death categories. Results are presented for broader age categories of <1, 1–4, 5–14, 15–49, and 50+ years age groups. Unknown, undetermined and missing causes of death in each sex and age category (7% of all deaths), were redistributed proportionately to other cause of death categories. The YLL were then calculated by multiplying the number of deaths in each subcategory of sex, age group and cause of death, by the Global Burden of Disease standard life expectancy at that age with 3% discounting, and an age weighting function of 0.04. Still births were included in this analysis and were given equal weight as deaths at age 0. This implies that the YLL loss from a stillbirth is valued the same way as that from death at age 0 and assumes the instantaneous rather than the gradual acquisition of life potential after 27 weeks of gestation [19].

## Results

### Descriptive characteristics

**Figure 1:**
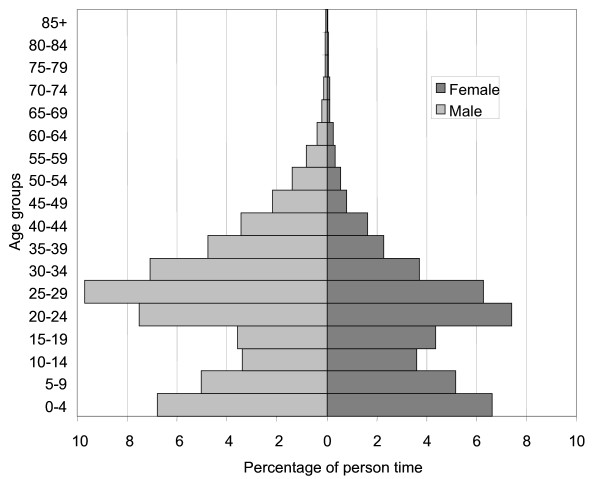
**Population pyramid for the study population, Nairobi DSS 2003–2005**. Light grey bars indicate the population distribution for males and dark grey bars indicate the distribution for females. Calculations are based on the observed person time contributed to each age group over the study period.

Over the study period residents in the DSA accumulated 162,505 person years. The mid-year population in 2003 was 56,479, in 2004 it was 54,442 and in 2005 it was 56,401. Given the size of the area (0.92 km^2^) this translates into a population density of about 60,000 people per km^2^. There were 5,301 live births and 1,346 deaths during the study period. The infant mortality rates (1q0) were 96.0, 82.6 and 81.8 per 1,000 live births for the years 2003, 2004 and 2005 respectively. The under-five mortality ratios (5q0) were 139.1, 119.1 and 121.4 per 1,000 live births respectively. Over the whole study period, the mortality rates for the other age groups were 1.7, 6.1 and 27.0 per 1,000 person years for the 5–14, 15–49 and 50+ years' age groups respectively. The study population has a peculiar population structure dominated by people in the productive ages (20–34 years), especially males and young children who are most likely their accompanying offspring, as shown in Figure 1.

### Burden of mortality estimates

**Figure 2:**
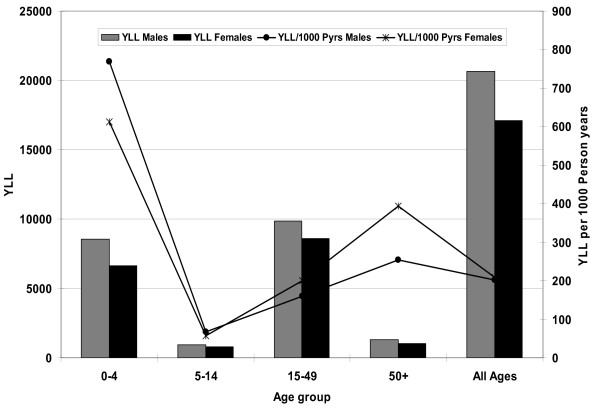
**Distribution of the premature mortality burden by age and sex expressed as YLL and YLL per 1,000 person years, Nairobi DSS, 2003–2005**. On the primary y-axis, light shaded bars are for male YLL and dark shaded bars are for female YLL. On the secondary y-axis, the line graph with star-shaped markers shows the YLL per 1,000 person years for females, while the one with round markers shows male YLL per 1,000 person years.

In the study population over the three year period, the mortality burden was 37,728 YLL. The distribution of YLL by age group, sex as well as for the whole study population is shown in Figure 2. The contribution to the overall burden by different age groups shows the expected relatively high burden among children under the age of five. The largest contribution comes from the 15–49 years age group, and minimal contribution from the elderly. This partly reflects the underlying population distribution. As shown in Figure 1, the 15–49 years age group contributes the largest proportion of person years. Maternal causes accounted for 754 YLL which is about 3.3% of the total YLL for the population aged five years and above, and 7.2% of YLL for females in the same age category.

Since a simple age distribution of the burden may be influenced by the underlying population structure, we also present the age shares of the mortality burden per capita expressed per 1,000 person years (Figure 2). As expected, the highest per capita burden is among children under five years old and the least is among the 5–14 year age group. The mortality burden among children under five (692/1,000 person years) is more than four times that in the rest of the population combined. This is despite the fact that the mortality burden in the population older than five years is rather high at 160 YLL per 1,000 person years. The mortality burden in the whole population is 205 YLL per 1,000 person years with minor sex differences. Males under five years, however, have a noticeable mortality disadvantage, while those aged 15–49 and 50+ years have a mortality advantage over females in the same age groups.

### Causes of the mortality burden

Based on the classification used in the initial BoD studies, the contributions of broad causes of death (Group I: communicable, perinatal, maternal and nutritional causes; Group II: non-communicable diseases and Group III: injuries) are shown in Table 1. Group I causes account for about 77% of the mortality burden while Group II causes account for the least mortality burden. Group III causes account for about 13% of the mortality burden in the whole population. There are some differences between the percentage shares of YLL by broad causes among the study population and the sub-Saharan Africa estimates for 2002. While the contribution of Group II causes is similar, the lower contribution of Group I causes is compensated for by the higher contribution of injuries. The percentage share of Group III causes in our study population is higher by 5 percentage points than the sub-Saharan African one.

**Table 1:** Premature mortality burden by broad causes of death in the Nairobi DSS, 2003–2005 compared with estimates in 2002 for sub-Saharan Africa

**Broad causes**	**YLL NUHDSS**	**% share**	YLL sub-Saharan Africa* (,000)	**% share**
Group I – Communicable Diseases. Maternal, Perinatal and Nutritional Causes	29143	77.2	230221	82.3
Group II – Noncommunicable Causes	3824	10.1	26843	9.7
Group III – Injuries	4761	12.6	20724	7.5

**Total**	**37727**	**100.0**	**277788**	**100.0**

* Source for sub-Saharan Africa estimates [31].

The main causes of the mortality burden among children aged less than five years and among the rest of the population are shown in Tables 2 and 3 respectively. Pneumonia, diarrhoeal diseases and stillbirths are the leading contributors accounting for close to 60% of the mortality burden among children under five. Among the rest of the population (5+ years), AIDS and tuberculosis combined are the leading contributors, accounting for about 50% of the mortality burden. AIDS and tuberculosis were combined in the analysis because about 35% of deaths from AIDS and tuberculosis were due to a probable combination of HIV/AIDS and TB, and hence were coded as "AIDS with TB," while seven cases were coded as "unspecified TB/AIDS." Interpersonal violence injuries (homicide) are the second most common contributor to the burden in the population aged five years and above, followed by road traffic accidents. Among the homicide deaths, gunshot wounds, and blunt trauma as a result of mob justice, are the most common modes of injury. Malaria accounts for only a small proportion of the mortality in both children under five (3.5%) and the population aged five years and above (1.9%).

**Table 2:** Top ten causes of premature mortality among children under the age of five years ranked by percentage contribution to the total YLL in the Nairobi DSS 2003–2005

**Causes**	**YLL**	**% YLL**	**Rank**
Pneumonia	3463	22.8	1
Diarrhoeal Diseases	2969	19.5	2
Stillbirths	2480	16.3	3
Malnutrition and Anaemia	1275	8.4	4
Birth Injury and/or Asphyxia	661	4.3	5
AIDS and TB	609	4.0	6
Malaria	537	3.5	7
Prematurity and or Low Birth Weight	529	3.5	8
Acute Febrile Illness	434	2.9	9
Measles	304	2.0	10

**Total (All Causes)**	**15192**		

**Table 3:** Top ten causes of premature mortality among the population aged five years and above ranked by percentage contribution to total YLL in the Nairobi DSS, 2003 – 2005

**Causes**	**YLL**	**% YLL**	**YLL Rank**
AIDS and Tuberculosis	11252	49.9	1
Interpersonal Violence Injuries	2719	12.1	2
Road Traffic Accidents	1302	5.8	3
Meningitis	781	3.5	4
Direct Maternal Causes	755	3.3	5
Pneumonia	524	2.3	6
Malaria	418	1.9	7
Cancer of the Gastrointestinal Tract	374	1.7	8
Renal Disorders	362	1.6	9
Malnutrition and Anaemia	305	1.4	10

**Total (All Causes)**	**22535**		

## Discussion

We analysed data collected by the longitudinal surveillance system of the NUHDSS which covers a population of about 56,000 residents in two slums. The population has a high disease burden especially among children under the age of five years. Pneumonia, diarrhoeal diseases and stillbirths account for more than half the burden among children under five, while HIV/AIDS, tuberculosis, interpersonal violence injuries and road traffic accidents account for more than two thirds of the burden among people aged five years and older.

The age structure of the study population is remarkably similar to the population structure of Nairobi province [20] and is characteristic of a predominantly labour migrant population which has more people in the productive age groups most likely accompanied by some of their younger offspring. The distribution of YLL by age group reflects this age structure. However, the large shares of the mortality burden borne by children under five and the 15–49 years age group reflect the effect of childhood illnesses, maternal causes and HIV/AIDS in these age groups. The underlying age structure is controlled for by calculating the age shares per capita or the mortality burden per 1,000 person years. This shows the tremendous mortality burden among children under the age of five years compared to the rest of the population, as well as the expected high burden among the elderly. The findings of a high mortality burden among the study population reinforce the findings from previous studies that have shown that the urban poor have poor health status, which in some instances, is worse than that of rural dwellers. These studies have however only shown this through high childhood mortality [10,11]. Since there are no similar estimates for other parts of Kenya, we compared our findings with estimates for the Tanzania coastal sentinel DSS site in 2003.

Estimates from the coastal DSS sentinel site in Tanzania using the same approach show a total per capita burden of 181 YLL/1,000 person years in 2003 (unpublished data) compared to 205 YLL/1,000 person years in our study population. Despite the fact that the burden in our population aged over five years is high (160 YLL/1,000 person years compared to 133 YLL/1,000 person years in the Tanzanian site), children under the age of five years still have more than four times the mortality burden of the rest of the population. In comparison, estimates from the DSS sentinel site in Tanzania show that children under five have about 3.6 times the burden of the rest of the population. Therefore, not only does our study population have a higher mortality burden than the Tanzanian DSS site, children in Nairobi slums have an even greater mortality disadvantage, compared to the rest of the population, than their Tanzanian counterparts. This disadvantage is also reflected in the infant and childhood mortality estimates from the Tanzania coastal DSS site, which are much lower that the estimates from our study population. In 2003, the infant mortality rate (1q0) in the coastal DSS site was 46.3, while the under-five mortality ratio (5q0) was 75.4 per 1,000 live births [21] compared to 96 and 139 respectively in our study population.

Perinatal causes account for 28% of under-five YLL which is rather high. According to estimates of global childhood mortality, in countries with high under-five mortality rates, neonatal causes account for about 20% of all childhood deaths [22,23]. Our study population is in a country with high childhood mortality (115 per 1,000 live births) [24], and estimates for the slums of Nairobi and the NUHDSS slums show an under-five mortality rate(151 and 139 per 1,000 live births respectively) higher than the national estimate [5]. The finding of a high proportion of neonatal deaths (33%, including stillbirths) therefore shows the increased vulnerability of children around the time of birth and is a reflection of the lack of good quality antenatal and delivery services in the study area [25]. We chose to include stillbirths in the YLL calculation in order to highlight their substantial contribution to the poor health status of the population.

The top ten causes of mortality among children under the age of five years in our study population are similar to the top ten causes of mortality for sub-Saharan Africa, but the order is different. Malaria plays a less significant role in the study area than in other parts of sub-Saharan Africa. This is because of the high altitude and cold climate in Nairobi which are unsuitable for malaria transmission. The small contribution of malaria is however in contrast to its position as the second most common diagnosis in health facilities in Nairobi province based on routine health information system reports [26], and the single most common self-reported illness in the study population [27].

The significant role played by pneumonia is unsurprising given the poor housing and overcrowding conditions in the slums. Another contributing factor may be the common practice of using the same room for cooking and sleeping coupled with the widespread use of kerosene for cooking (APHRC, unpublished data). A morbidity survey in 2000 showed that 46% of children aged below 36 months living in the slums of Nairobi had had cough in the two weeks preceding the survey, while 32% had had diarrhoea [28]. Our findings therefore reinforce those from the morbidity survey which show the relatively high importance of these two conditions in influencing the health of slum children. On the other hand, given the high mortality burden from AIDS in the adult population, it is possible that some paediatric HIV-related deaths may be misclassified as diarrhoeal diseases or pneumonia, as has been the case in other studies [29]. In most cases, there are no death certificates to complement the oral interview. Most people die outside the formal health care system and the tool does not capture HIV status in any other way except what is volunteered by the respondent. The verbal autopsy approach therefore has limitations in this case and is unable to capture the likely high proportion of AIDS-related deaths.

Malnutrition contributes less than 10% of the YLL among children under the age of five years. This may be because, in the verbal autopsy approach, respondents are more likely to report the more dramatic events surrounding the death of a child such as diarrhoea and cough, rather than the more insidious underlying malnutrition. It is therefore likely that the malnutrition burden is underestimated in the study population since it is known that malnutrition is an underlying cause for more than half of childhood deaths. Given the levels of poverty and deprivation in the slums, one would expect malnutrition to play a more significant role than we found. On the other hand, although malnutrition may be prevalent, it may not necessarily contribute directly to mortality since chronic types of malnutrition are rarely fatal.

Among the population aged 5+ years and above, the devastating effect of the HIV/AIDS epidemic is evident in the large contribution of AIDS and tuberculosis to the mortality burden. As previously indicated in the results section, these two illnesses were combined in the analysis because close to one third of deaths from these two causes could be attributed to both conditions, i.e. the code "AIDS and Pulmonary TB" and some deaths (seven) were classified as "unspecified AIDS/TB." It is also known that most TB cases in sub-Saharan Africa are related to underlying HIV/AIDS and that TB is a common opportunistic infection in HIV-related deaths in Nairobi [30]. Teasing one from the other without additional information such as medical records is difficult, hence the decision to combine the two. While this masks the role of other opportunistic infections, it still highlights the tremendous impact which both conditions have on the study population. Estimates from sub-Saharan Africa show that these two conditions contribute only about 30% of the YLL burden [31]. While it is possible that the YLL burden may have increased since 2002 when these estimates were calculated, our study suggests that slum residents in Nairobi have been hit by the HIV/AIDS epidemic in perhaps greater terms than other subpopulations in sub-Saharan Africa.

Injuries due to interpersonal violence are the second leading contributor to the mortality burden in the population aged five years and older. The contribution of all injuries to the overall burden in the whole population is also higher than in sub-Saharan Africa. The most common modes of injury are gunshot wounds, road traffic accidents and blunt trauma during mob justice. The modes of injury reflect the high levels of insecurity and violence in the population. Informal settlements are characterized by living and social conditions which are known risk factors for violence. At the society level, they include reduced inhibitions against violence, and the creation and sustenance of gaps between different segments of society [32]. At the community level, social disintegration, high residential mobility, high population density, heterogeneity and lack of social cohesion play a role. At the individual level, low educational attainment, drug and alcohol abuse, and unemployment are important [32]. Most, if not all, of these factors are present in informal settlements and hence the high level of interpersonal violence is unsurprising. Additionally, the marginalization of residents in informal settlements means that they are rarely served by social institutions such as the police and justice system. Perceived indifference of such institutions to injustice in slum communities often results in community measures which usually take the form of "mob justice" meted out to people suspected of committing crimes, even petty ones.

The high contribution of road traffic accidents to the mortality burden reflects the growing importance of this cause in sub-Saharan Africa [33], as well as the effect of rapid urbanization. Growing traffic volumes accompanied by poorly planned cities, poor state of roads and ineffective law enforcement are known factors which contribute to the increasing burden of road traffic accidents in sub-Saharan Africa, and Nairobi is no exception.

Our study's strength is in its longitudinal nature which enabled us to follow up a large population, record deaths soon after they occurred, have an accurate reference population and the time at risk, and conduct verbal autopsies in the majority of cases. We were therefore able to calculate both the overall mortality burden and the age-specific burden per capita which identifies subgroups at higher risk than others.

The verbal autopsy approach fills the gap left by the absence of vital registration systems and other cause of death ascertainment mechanisms despite its shortcomings. Since the majority of deaths occur outside the formal health sector, we could not use official death certificates, more so since the quality of the existing system in Kenya is unknown.

The sensitivity of the verbal autopsy is however dependent on how prevalent a certain disease is [34]. Causes of death like malaria and AIDS are more likely to be assigned by coders in areas with a high prevalence of the illness compared to coders in areas with low prevalence. Conversely the same causes are less likely to be assigned where the prevalence is truly or perceived to be low. The sensitivity of the verbal autopsy also depends on how distinct a disease's signs and symptoms are compared to other diseases. Diseases with common symptoms such as fever may be more frequently misclassified compared to those with distinct symptoms such as a stiff neck [17]. Misclassification errors are even more common in coding childhood deaths. This is because children often have more than one illness and it may be difficult to determine which of these the main cause of death is. While these misclassification errors may not affect the overall YLL estimates, they may lead to underestimation of YLL contributed by specific causes. Our inability to assign causes of death in about 7% of deaths is a limitation of the study which was mitigated by redistributing these deaths to other cause of death categories.

## Conclusion

The high disease burden among slum residents, especially among children under the age of five years, the high burden of fatal injuries, and the devastating effect of HIV/AIDS in a context of rapid growth of urban settlements and high proportion of poor people in these settlements raise serious challenges for governments in sub-Saharan Africa. Large subpopulations with high disease burden may threaten progress towards national and global development goals such as the Millennium Development Goals, especially those aimed at reducing childhood mortality and the impact of the HIV/AIDS epidemic, unless drastic steps are taken to address the health and social needs of the urban poor. The successful utilization of a BoD approach for district and national planning in Tanzania and its impact on child mortality offer a way out for marginalized slum dwellers in Nairobi City. Based on data from the NUHDSS, health planners could adopt a similar planning approach that would benefit slum dwellers.

## Competing interests

The author(s) declare that they have no competing interests.

## Authors' contributions

CK did the data analysis, wrote the first draft of the manuscript and contributed to the interpretation of the results. AKZ collected the data, participated in coding the verbal autopsies, contributed to the data analysis, writing of the paper and interpretation of the findings. YY did the literature review, contributed to the writing of the paper and interpretation of the findings. AE conceptualized the NUHDSS, contributed to the writing of the paper and the interpretation of the findings. All authors read and approved the final manuscript

## References

[B1] UN-Population Division (2002). World Urbanization Prospects: The 2001 Revision.

[B2] United Nations Human Settlement Programme (2003). Slums of the World: The face of Urban Poverty in the New Millennium?.

[B3] Lamba D (1994). The forgotten half; environmental health in Nairobi's poverty areas. Environ Urban.

[B4] Todaro M (1989). Urbanization and rural-urban migration: Theory and policy. Economic Development in the Third World.

[B5] African Population and Health Research Centre (2002). Population and Health Dynamics in Nairobi Informal Settlements Nairobi.

[B6] Magadi MA, Zulu EM, Brockerhoff M (2003). The inequality of maternal health care in urban sub-Saharan Africa in the 1990s. Popul Stud (Camb).

[B7] Brockerhoff M, Brennan E (1998). The poverty of cities in developing countries. Popul Dev Rev.

[B8] Hacker A, Ryan C (2003). Prevalence of infant stunting in an urban Kenyan population: comparison to the 1998 Kenyan Health and Demographic Survey and the 2000 CDC growth grids. Nutr Res.

[B9] Taffa N (2003). A comparison of pregnancy and child health outcomes between teenage and adult mothers in the slums of Nairobi, Kenya. Int J Adolesc Med Health.

[B10] Madise NJ, Banda EM, Benaya KW (2003). Infant mortality in Zambia: socioeconomic and demographic correlates. Soc Biol.

[B11] Madise NJ, Diamond I (1995). Determinants of infant mortality in Malawi: an analysis to control for death clustering within families. J Biosoc Sci.

[B12] Murray CJ, Lopez AD (1997). Regional patterns of disability-free life expectancy and disability-adjusted life expectancy: Global Burden of Disease study. Lancet.

[B13] Murray CJ, Lopez AD (1996). Global Burden of Disease: A Comprehensive Assessment of Mortality and Disability from Diseases, Injuries, and Risk Factors in 1990 and Projected to 2020 (The Global Burden of Disease and Injury).

[B14] Murray CJ, Lopez AD (1997). Mortality by cause for eight regions of the world: Global Burden of Disease Study. Lancet.

[B15] Wurthwein R, Gbangou A, Sauerborn R, Schmidt CM (2001). Measuring the local burden of disease. A study of years of life lost in sub-Saharan Africa. Int J Epidemiol.

[B16] de Savigny D, Kasale H, Mbuya C, Reid G (2005). In focus: Fixing Health Systems.

[B17] Anker M, Black RE, Coldham C, Kalter HD, Quigley MA, Ross D, Snow RW (1999). A Standard Verbal Autopsy Method for Investigating Causes of Death in Infants and Children.

[B18] Soleman N, Chandramohan D, Shibuya K (2006). Verbal autopsy: current practices and challenges. Bull World Health Organ.

[B19] Jamison DT, Shahid-Salles SA, Jamison J, Lawn JE, Zupan J, Lopez AD, Mathers CD, Ezzati M, Murray CJL, Jamison DT (2006). Incorporating Deaths near the Time of Birth into Estimates of the Global Burden of Disease. Global Burden of Disease and Risk Factors.

[B20] Government of Kenya (2001). The 1999 Population and Housing Census.

[B21] Tanzania Ministry of Health (2005). District Health Interventions Profile 2004: Rural coastal districts.

[B22] Jones G, Steketee RW, Black RE, Bhutta ZA, Morris SS (2003). How many child deaths can we prevent this year?. Lancet.

[B23] Black RE, Morris SS, Bryce J (2003). Where and why are 10 million children dying every year?. Lancet.

[B24] CBS, MoH, ORC Macro (2005). Kenya Demographic and Health Survey 2003 Calverton, Maryland.

[B25] African Population and Health Research Centre (2007). Averting Preventable Maternal Mortality: Delays and Barriers to the Utilization of Emergency Obstetric Care in Kenya, Country Report.

[B26] Government of Kenya – Ministry of Health (1999). Epidemiological Trends.

[B27] Ye Y, Kimani E, Kebaso J, Mugisha F (2007). Assessing the risk of self-diagnosed malaria in urban informal settlements using self-reported morbidity survey. Malar J.

[B28] African Population and Health Research Center (2002). Population and health dynamics in Nairobi Informal Settlements.

[B29] Groenewald P, Nannan N, Bourne D, Laubscher R, Bradshaw D (2005). Identifying deaths from AIDS in South Africa. AIDS.

[B30] Rana FS, Hawken MP, Mwachari C, Bhatt SM, Abdullah F, Ng'ang'a LW, Power C, Githui WA, Porter JD, Lucas SB (2000). Autopsy study of HIV-1-positive and HIV-1-negative adult medical patients in Nairobi, Kenya. J Acquir Immune Defic Syndr.

[B31] World Health Organisation (2003). Revised Global Burden of Disease (GBD) 2002 Estimates Geneva.

[B32] Krug EG, Dahlberg LL, Mercy JA, Zwi AB, Lozano R, World Health Organisation (2002). World report on violence and health.

[B33] World Health Organisation (2002). Injury: A leading cause of the global burden of disease, 2000.

[B34] Setel PW, Rao C, Hemed Y, Whiting DR, Yang G, Chandramohan D, Alberti KG, Lopez AD (2006). Core Verbal Autopsy Procedures with Comparative Validation Results from Two Countries. PLoS Med.

